# P37 The effectiveness of a new short regimen for drug-sensitive TB: preliminary results

**DOI:** 10.1093/jacamr/dlaf230.044

**Published:** 2025-12-04

**Authors:** V Guseva, I Vasilyeva, V Testov

**Affiliations:** National Medical Research Center of Phthisiopulmonology and Infectious Diseases, Moscow, Russian Federation; National Medical Research Center of Phthisiopulmonology and Infectious Diseases, Moscow, Russian Federation; National Medical Research Center of Phthisiopulmonology and Infectious Diseases, Moscow, Russian Federation

## Abstract

**Background:**

In accordance with the 2022 recommendations of the WHO and the 2024 National Guidelines of the Russian Federation, alongside the standard 6 month chemotherapy regimen for drug-sensitive TB (DS-TB), a 4 month regimen comprising rifapentine (1200 mg/daily) instead of rifampicin, and moxifloxacin instead of ethambutol, is permissible. However, the use of this regimen in clinical practice may be limited by the high dose of rifapentine, particularly in patients with comorbidities. Therefore, developing alternative shortened therapy regimens with a favourable safety profile and proven efficacy remains an urgent task.

**Objective:**

To compare the effectiveness of treatment of pulmonary TB with preserved susceptibility of *M. tuberculosis* to isoniazid, rifampicin and fluoroquinolones between the standard 6 month regimen and the new short 4 month regimen with rifapentine (900 mg/daily) instead of rifampicin and levofloxacin instead of ethambutol.

**Methods:**

The treatment of 92 TB patients with preserved drug susceptibility to anti-TB drugs was analysed. The patients were divided into two equal groups that were comparable in terms of age, gender and prevalence of the disease. The main group underwent a 4 month regimen comprising an intensive phase (8 weeks) of rifapentine (900 mg/daily), isoniazid, pyrazinamide and levofloxacin, followed by a continuation phase (9 weeks) of rifapentine (900 mg/daily), isoniazid and levofloxacin. The control group was assigned a standard 6 month schedule. The preliminary effectiveness of the 4 month regimen was assessed using a cumulative score determined at the primary combined endpoint (8 weeks), according to the following criteria: sputum culture conversion, positive X-ray dynamics and improvement in the intoxication syndrome.

**Results:**

The cumulative score at the primary combined endpoint (8 weeks) was 34.89 ± 3.2 in the 4 month regimen group and 25.56 ± 1.5 in the control group (Student’s *t*-test, *P*=0.019).

**Conclusions:**

Preliminary results indicate that a 4 month regimen involving rifapentine (900 mg/daily), isoniazid, pyrazinamide and levofloxacin is more effective than a 6 month regimen for treating DS-TB.

